# Supplementary figures and images for: Spatiotemporal inflection points in human running: Effects of training level and athletic modality
Source: PLoS One. 2021 Oct 18;16(10):e0258709. doi: 10.1371/journal.pone.0258709 (PMC8523042; doi:10.1371/journal.pone.0258709)

# Sprinters

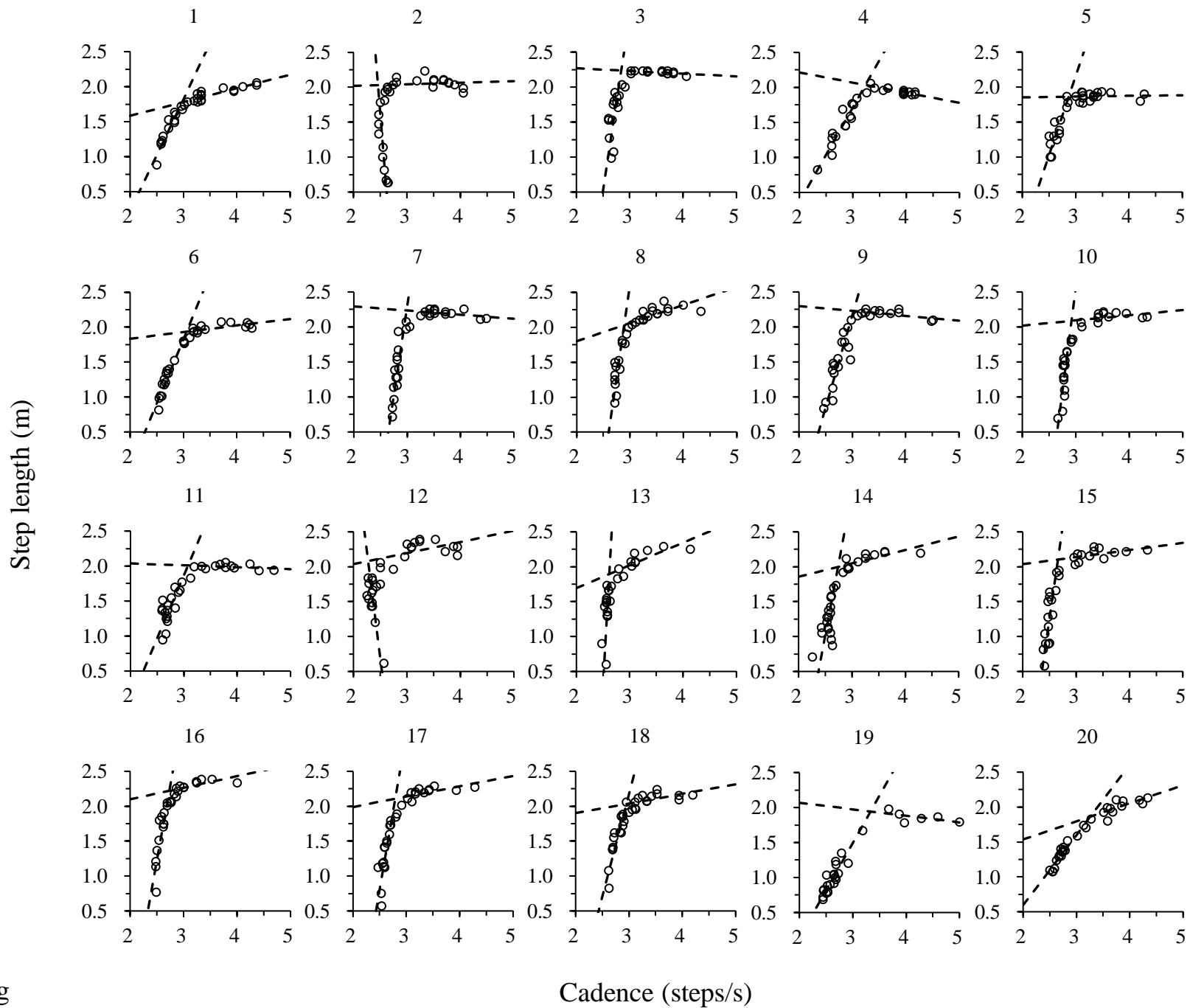

S1 Fig

Supplement: S1 Fig — The two dashed lines depict the regression lines computed from different data below and above the inflection point, respectively. (PDF) [file pone.0258709.s001.pdf]

# Distance runners

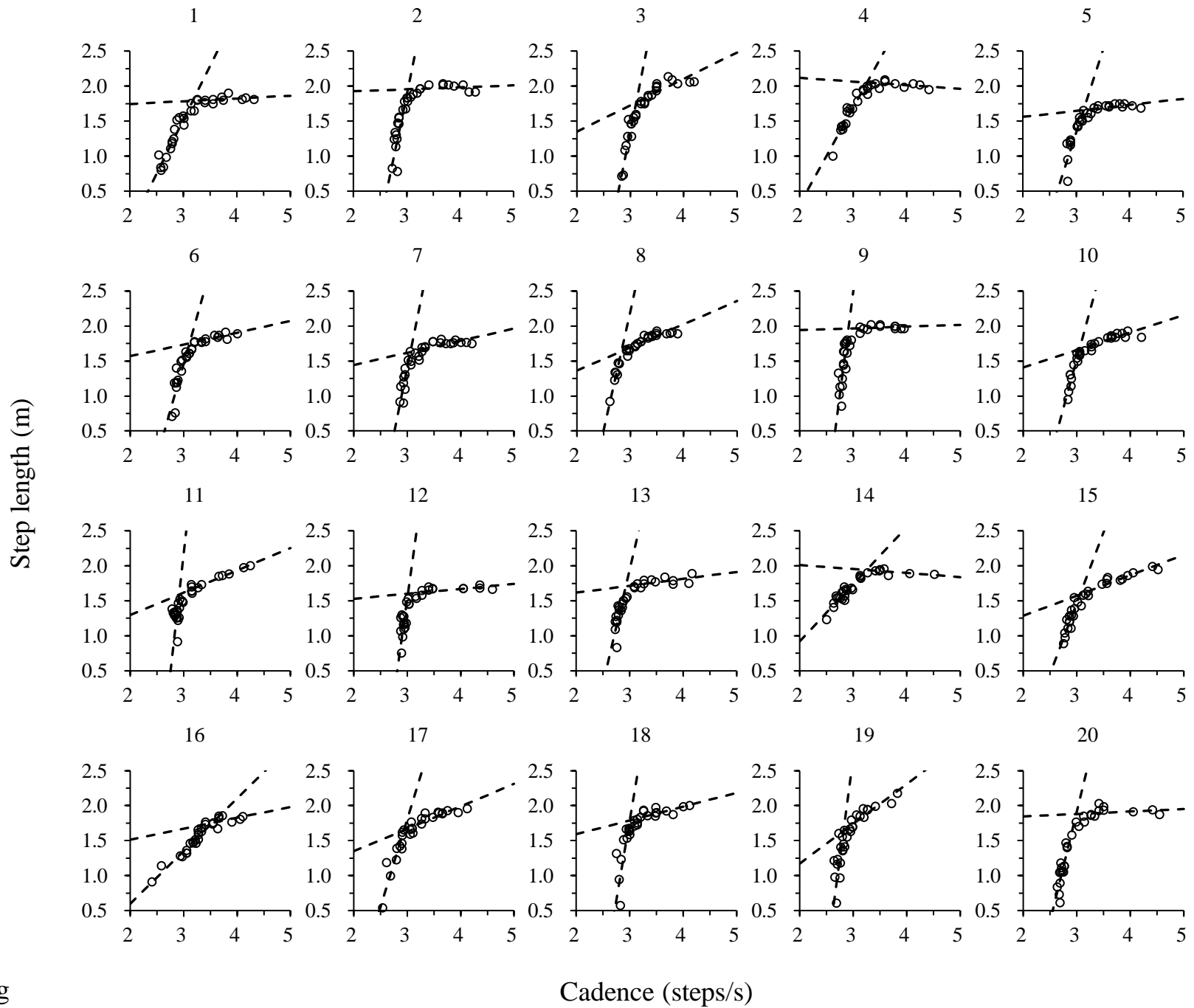

Supplement: S2 Fig — The two dashed lines show the regression lines computed from different data below and above the inflection point, respectively. (PDF) [file pone.0258709.s002.pdf]

# Active athletes

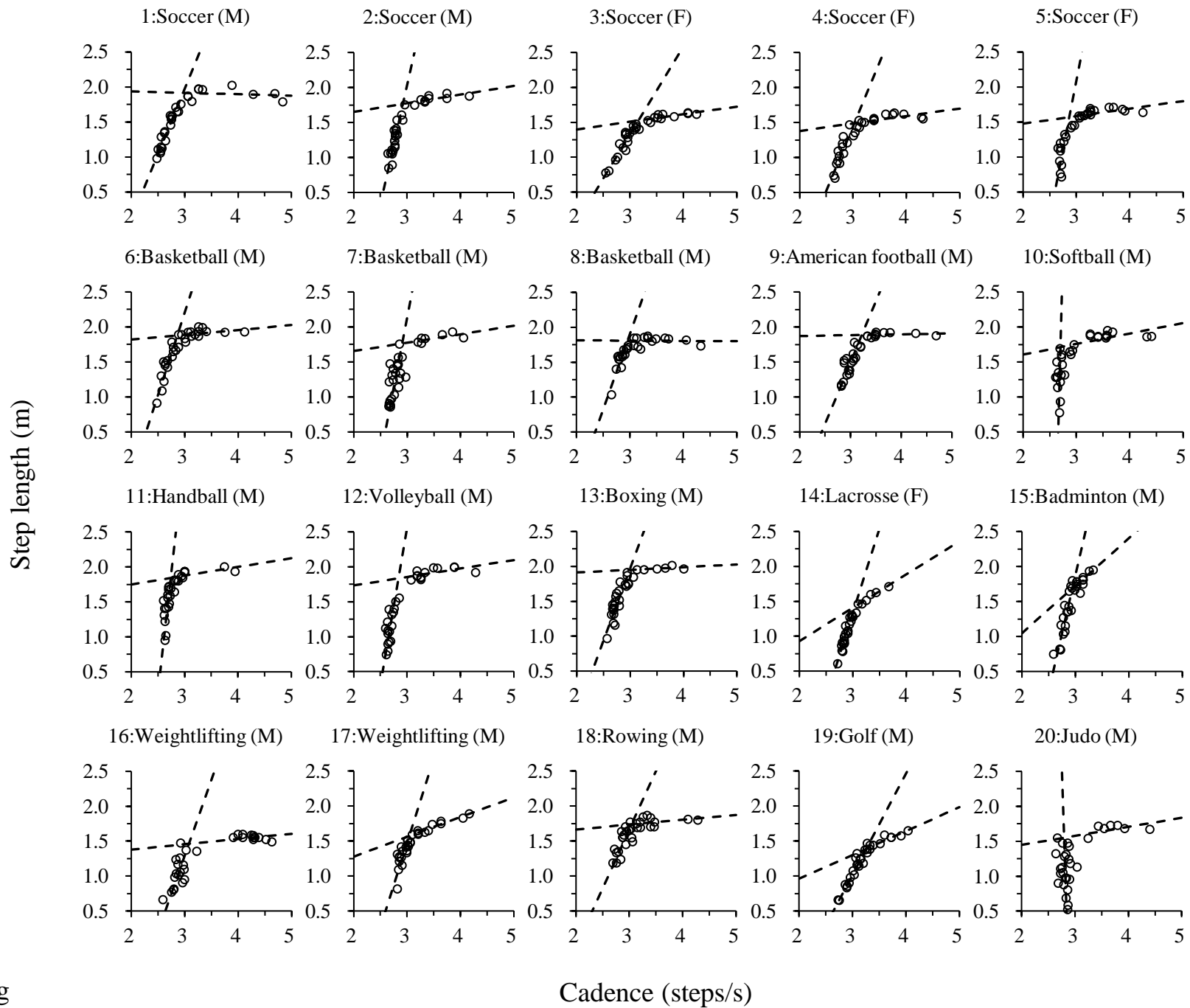

S3 Fig

Supplement: S3 Fig — The two dashed lines show the regression lines computed from different data below and above the inflection point, respectively. The title of each figure corresponds to each subject’s sports experience. Characters in parentheses signify male or female subjects. (PDF) [file pone.0258709.s003.pdf]

# Sedentary

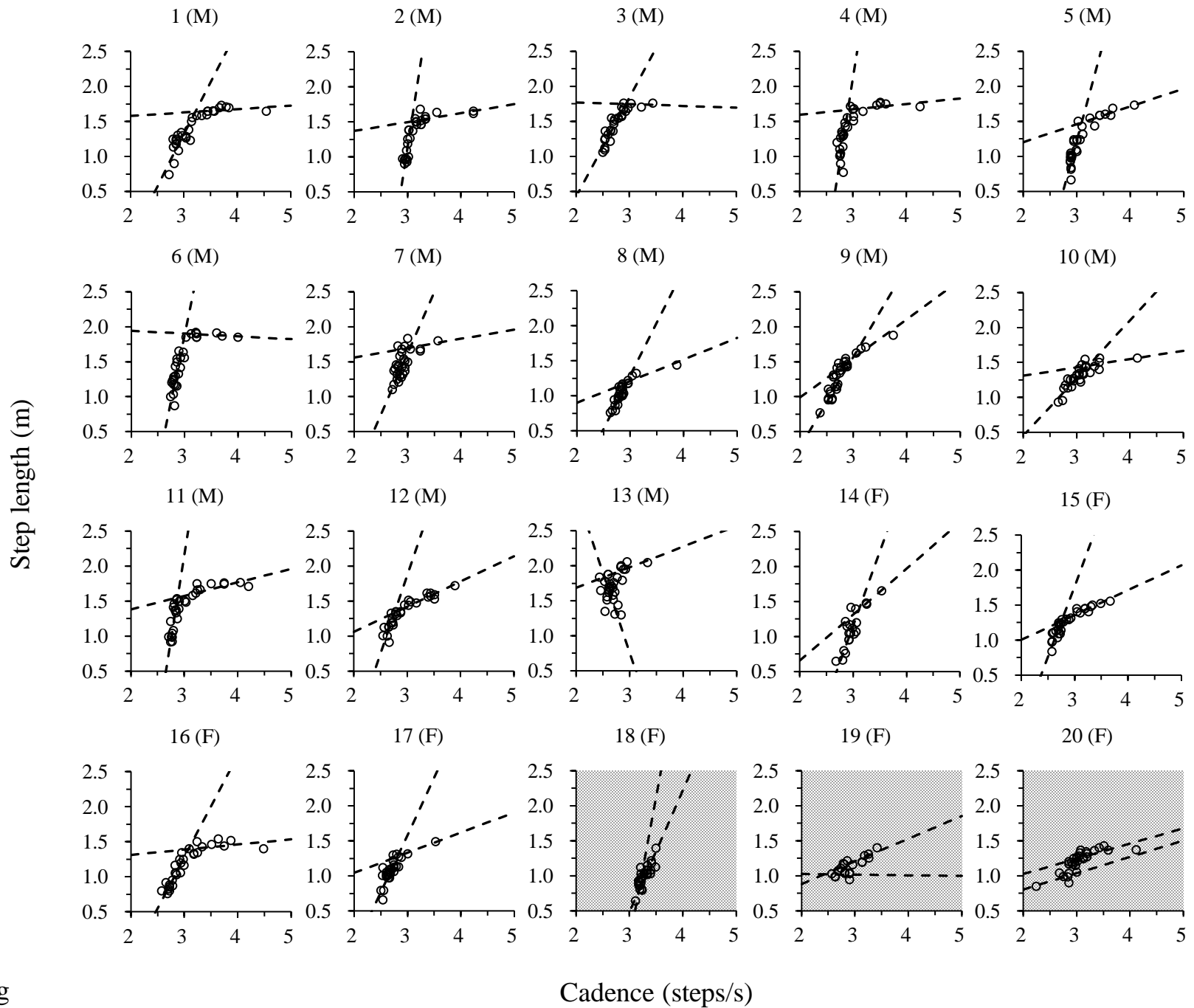

S4 Fig

Supplement: S4 Fig — The two dashed lines show the regression lines computed from different data below and above the inflection point, respectively. In the sedentary group, three subjects were excluded from data analysis: two subjects (No. 18 and No. 19) had estimated inflection point fell outside the range of the original data, and one subject (No. 20) showed two regression lines with almost the same slope giving the inflection point completely outside the range of measured data. Characters in parentheses signify male or female subjects. (PDF) [file pone.0258709.s004.pdf]
