# Supplementary material for: Spatiotemporal inflection points in human running: Effects of training level and athletic modality
Source: PLoS One. 2021 Oct 18;16(10):e0258709. doi: 10.1371/journal.pone.0258709 (PMC8523042; doi:10.1371/journal.pone.0258709)
Supplement: Attachment — Submitted filename: respone to reviewers_PONE-D-21-04779_Goto.docx [file pone.0258709.s005.docx]

- **Reviewer #1: Major questions**
  General observations
  The study presents relevant results to the Sports Science and for the area of human locomotion from a biological/evolutionary point of view, such as the fact that stride frequency is similar at the inflection point between groups (an innate pattern). In this sense, I suggest some observations:
  We appreciate the positive evaluation of our paper. We have revised the manuscript according to your and other reviewers’ suggestions. We agree that the former version contained too many speculative statements and also that the structure was too complicated. Therefore, we deleted Fig. 2, showing the V-C-S curve of all subjects in one picture, because the basic messages overlap with Fig. 3 as well as the supplementary figures. We added Table 2 to provide numerical data concerning the inflection point. Finally, we changed the order of Fig. 3 (new Fig. 3) and Fig. 4 (new Fig. 2). We hope that, relative to the old version, it has been satisfactorily revised and the logic is clearer and more straightforward.

- to modify the title to include some aspect of the result from a broader view and not just Sports Science

We appreciate the suggestion. Another reviewer also suggested changing the title. We modified it to “Spatiotemporal inflection points in human running: effects of training level and athletic modality”.

- in the introduction, this aspect needs to be considered in the justification / objectives / hypothesis (see line 13-14 of abstract: Since the influence of training on the basic V-C-S relationship is minimal, the basic pattern is largely innate). For example: What is this innate pattern??
We have tried to modify the manuscript so as to make the logic clearer, stating the hypothesis as: “the running characteristic of individuals should be influenced by the requirements of the primary sport they engaged in, as well as that the distance runners would exhibit the greatest tendency to change velocity by altering step length in the lower velocity range”. (ll. 54 - 57)

- in the discussion, it is possible to intensify these questions by adding other arguments. For exemple: in Line 383, it was not clear, what are these neural mechanisms? What actually happens (brief) ? Another issue is that these neural mechanisms may be a physiological effect of an underlying mechanical mechanism (Line 461): the relationship between forward work (Wf) and vertical work (Wv) of center of mass and its effects on internal work - Wint (linked to lower limb movement) vs external work – Wext (linked to stride length). Slower running speeds Wext > Wint // faster running speeds Wext < Wint . Intersection speed (beetwen Wext and Wint) can match with critical point velocity (???) (see Cavagna et al. 2008 – Fig 3. (DOI: 10.1098/rspb.2007.1288))// and stride frequency effects on mechanics: Minetti and Saibene 1992 Mechanical work rate minimization and freely chosen stride frequency (SF) of human walking: a mathematical model // Minetti and Alexander 1997: A Theory of Metabolic Costs for Bipedal Gaits. // That is, innate factors need to be better discussed, or else to assume as limitations of the discussion
We appreciate the very constructive suggestion. Yes, we are also very interested in the “why and how” for the differences in various parameters of the V-C-S relationship. Especially, the neural as well as physiomechanical mechanisms will be our main focus. However, this study is one step before that, and in this study we only analyzed whether or not there is any difference in the V-C-S relationship among different groups of subjects having different backgrounds of running training. If any, then we planned to advance to the study of analyzing the mechanism underlying it. We have not done any physiological or biomechanical measurement in this study; we just focused on velocity, SF, and SL. Of course we have hypotheses regarding the mechanism. However, they are no more than mere speculations, because we have no available data yet. Therefore, we decided not to mention the neural or physiomechanical hypothetical mechanisms in this paper, and we hope the reviewer will understand our reasoning. We did briefly mention our future intended direction in the “limitations of the present study” section (ll. 381 – 394).

Methods
- It is necessary to make it clear how many times the same speed was performed, in addition, describe exactly how many speeds in relation to the maximum (%) were performed (Were there 10 differents speeds? And, How many times each?)

The instruction was mainly given to subjects based on their own subjective effort. Therefore, there is almost no identical running speed in each subject's trial. The purpose of this method is to scatter data over a wide range of speeds, and we think this was accomplished as can be seen in the individual data of the supplementary figures (S1-S4).

- Two different criteria were used to determine the time over the path? line 128 and 131. Please, to clarify and determine which of the two ways was used to determine the time.
They are the times to calculate velocity and cadence, respectively. We added an explanation to make this point clearer (ll. 114 – 117).

- line 185: Variables except for running velocity and step length at maximal running velocity, and running velocity and step length at the inflection point exhibited non-normal distributions. Wouldn't it be better to describe which variables did not show normality?

We explained which variables had normal distributions, and which had non-normal distributions (ll. 157 - 159, ll.167 - 172).

Results
- A correlation of the spatiotemporal parameters was performed at Vmax. Why it was not done at the critical point speed? I think it would be really good point.
Vmax is a definite parameter used as a performance indicator. We actually made the correlation between the velocity at the inflection point and the spatiotemporal parameters. However, we can't explain any factors to explain the obtained results, because each parameter is a value from the same data point (inflection point). Therefore, we adopted “Vmax” as the independent variable for most of the analyses.

Discuss
- Important issues to highlight in the discussion:
Why SF is the same for all groups at the critical point velocity? Why around 60-65% of SF máx? Why stride length is different? At Vmax all groups were at maximum SL and SF, at critical point velocity not. The training history influenced SF and SL (futher) at maximum speed.
We appreciate the suggestion. We are also interested in why SF at the inflection point is similar among different groups of subjects. However, we are not able to explain the mechanism underlying this similarity from the data of this study. SL at the inflection point does not differ so much from the SL at Vmax, because the SL is already close to the limit at the inflection point. As for training history, it is necessary for sprinters to increase SF from the inflection point in order to obtain a higher maximum velocity. We have totally modified the Discussion so that the above points could be clearly understood.

Line 314-316: it seems that the reasons for removing subjects 18 and 19 in the discussion were not the same as the results and methods line 222-224: “In addition, the data for two subjects showed no clear inflection point (sedentary subjects No. 18 and No. 19).”
We apologize for the confusing description. We have modified the part so it now reads: “Subjects with inflection points, thus obtained, that differed largely from the measured points, were excluded from the analysis (#18, #19, and #20, as seen in Figure S4)” (ll. 148 – 149).

Line 355: “Relative step length, however, still had a positive correlation with Vmax (Fig. 5F); the higher the Vmax, the higher the relative step length at the inflection point. In particular, in the range below the inflection point, subjects in the higher Vmax range (sprinters) showed a relative step length of close to 100%, indicating that they increased step length almost to the limit.” I don’t agree with that interpretation. In the first place, this correlation was weak (such as the speed at the inflection point) and it represents that regardless of the training history, all groups had a similar relative SL and quite close to the maximum SL.
We appreciate the comment. In the revised manuscript, we emphasized that “However, this correlation was weak when it is normalized with the step length at the Vmax (Fig. 4F). Therefore, regardless of the training history, all groups had a similar relative step length quite close to the maximum step length (about 90%).”, as suggested by the reviewer, rather than mentioning a weak positive correlation (ll. 313-315).

Line 360: “This indicates that the higher the Vmax, the greater the scope for cadence increase in the velocity range above the inflection point.” It means that the history of the training influenced SF relative (not absolute) at the inflection point, that is, sprinters had at the inflection point a SF relative to the vmax lower than the others, although in absolute terms SF was the same, this interpretation was lacking.
We appreciate the comment. Yes, the higher the Vmax, especially in sprinters, the larger the cadence change from the inflection point to Vmax. We added “The history of the training influenced normalized cadence at the inflection point, that is, sprinters had a lower normalized cadence at the inflection point than the others, although in absolute terms cadence was the same.” (ll. 317 – 319).

Line 412 “Higher maximal velocity could be obtained with more efficient movements (running economy), and, thus, sprinters inevitably and unintentionally would pursue efficient movements.” I think the sentence should be written backwards due to the logic of the text and theoretical logic: runners running at a higher speed improve the efficiency and not the other way. Higher velocity needs more power and not economy. Not only economy but also…)

Thank you for the suggestion. We think that this is the most important point of this study. Of course, sprinters are pursuing a higher Vmax. But it cannot be accomplished only with power, especially at the highest levels. They would have to have both power and economy as they neared the limit. Therefore, sprinters would be expected to have the most efficient movement among the four groups, showing the highest absolute (Fig. 4C) and relative step length at the inflection point (Fig. 4F).

We have modified this part to read as: “However, sprinters and not distance runners obtained velocity by elongating step length, in both absolute step length (Figure 4C) and in relative step length (Figure 4F), all the way to the upper limit. Thus, our working hypothesis was rejected. Sprinters rarely train in the velocity range below the inflection point. Needless to say, maximal velocity is crucial for sprinters. A higher velocity cannot be accomplished only with power, especially at the highest levels. It might be possible that sprinters need to maintain both power and economy to the upper limit, and inevitably and unintentionally develop mechanically efficient movements. Therefore, the present results suggest that sprinters have the most efficient mechanical movement among the four groups, showing the highest step length at the inflection point.” (ll. 346 - 356) We hope that the part is now more understandable.

- line 474: “Quantitative differences in the various parameters of the V-C-S relationship across the groups likely resulted from differences in innate ability and/or different running backgrounds.” I think it is necessary to highlight in the conclusion which parameters would be attributed to innate factors and which factors to running background, for example, speed running and SF at critical point (innate) and vmax (background).

We appreciate the suggestion. In the revised manuscript we tried to exclude speculative description as much as possible. In fact, even though the inflection point is constant among different groups, it might be a result of adaptation commonly occurring along the development. Accordingly, we have omitted the word “innate”, and revised this part of the manuscript (ll. 396 - 407).

Minor questions
- 1Graduate school of Sport Sciences: capital letter – school

It was corrected.

- Line 446: extra space

It was corrected.

- **Reviewer #2: Reviewers’ comments:**
  The purpose of the present study was to reveal how a change in running velocity altered the spatiotemporal adjustment between step length and cadence in subjects with different histories of engagement in running training. The main finding was that quantitative differences in the various parameters of the velocity-cadence-step length relationship across the groups likely resulted from differences in innate ability and/or different running backgrounds. The topic of this study is interesting! However, methodological and structural aspects compromise this submission. Below, some comments to improve the manuscript for that or future submission.
  We appreciate the reviewer’s interest in this study. We have revised the manuscript according to your and other reviewers’ suggestions. We agree that the former version contained too many speculative descriptions and also that the structure was too complicated. Therefore, we deleted Fig. 2, which showed the V-C-S curve of all subjects in one diagram, because the basic messages overlap with Fig. 3 and the supplementary figures. We added Table 2 to provide numerical data concerning the inflection point. Finally, we changed the order of Fig. 3 (new Fig. 3) and Fig. 4 (new Fig. 2). We hope that it has been satisfactorily revised and, relative to the old version, the logic has become clearer and more straightforward.

Keywords: It is suggested to adopt keywords that are not written in the title.

Abstract: The summary is apparently speculative. I suggest inform the numerical results and base the conclusion on the independent variables adopted.

We have modified the summary so as to be minimally speculative, as seen in our revision: “In spite of the wide range of athletic modalities of the subjects, as well as their maximum running velocities, the inflection point appeared at a similar cadence (3.0 ± 0.2 steps/s) and at a similar relative velocity (65-70%Vmax), across all groups. These results imply that the influence of running-specific training on the inflection point is minimal”. We also tried to include more numerical results, but due to the word limit, we were not able to do so. (Please see below.)

Page 02, lines 04-06: Why the sedentary group ran 20m?
The running distance of the sedentary group was set to 20m, 10 m shorter than other groups, in consideration of their lack of physical strength and endurance. We omitted the distance from the Abstract, because it might have led to a misunderstanding, we did explain it in detail in the Method section (ll. 86 - 87).

Page 02, line 09: The running velocity changed, but how did these changes occur in each group?

We apologize for not making this part easier to understand. This sentence described the phenomenon of the V-C-S relationship. We omitted “as running velocity changed”, and tried to provide a comprehensible explanation of the V-C-S relationship (ll. 12 - 17). We hope our revised explanation is now more understandable.

Page 02, lines 11-12: In fact, changes in running speed occur due to changes in length and then stride frequency (Nummela, et al., 2007).
We added the suggested reference to the abstract (l. 15).

Page 02, lines 12-14: Impossible to understand this sentence. Influence of training? Is this a variable independent of this study? I suggest removing this sentence.
This sentence was too speculative and we therefore removed it.

Page 02, lines 16-17: I suggest inform the average values and standard deviations of running speeds, stride lengths and stride frequencies of each group.
Thank you for the suggestion. The data you mention are very informative but too extensive to be included in the Abstract. If it were done, the number of words would exceed the limit (300 words). Since we could not do this, we did add Table 2 to make the data available.

Page 02, line 17: This cannot be said because no training has been investigated.
The corresponding sentence was deleted.

Page 02, lines 18-20: This conclusion is apparently speculative. Based in this abstract, it is not possible to conclude that, for example, the training history does not affect than cadence.
We appreciate the suggestion. We have modified the last part of the Abstract so that it is not speculative. It now reads: “In spite of the wide range of athletic modalities of the subjects, as well as their maximum running velocities, the inflection point appeared at a similar cadence (3.0 ± 0.2 steps/s) and at a similar relative velocity (65-70%Vmax), across all groups. These results imply that the influence of running-specific training on the inflection point is minimal “. (ll. 19 - 23)

Introduction: The introduction is pertinent. However, the study's justification is not convincing. A more specific approach is suggested regarding the physiomechanical mechanisms of running (see, Minetti, A. E., for example). There is a need to adopt new bibliographic references. Finally, parts of the structure correspond to the methodology.
We appreciate the constructive suggestion. Yes, we are also very interested in why and how the differences in various parameters in the V-C-S relationship come from. Especially, the physiomehcanical mechanisms are our main focus. But this study is one step before that, and we just analyzed whether or not there is any difference in the V-C-S relationship among different groups of subjects having different background of running training. If any, then we planned to advance to the study of analyzing the mechanism underlying it. We have not done any physiological or biomechanical measurement in this study, just focusing on velocity, SF, and SL. Of course we have hypotheses on the mechanism. However, they are no more than mere speculation, because we have no available data yet. Therefore, we decided not go deeper into the physiomechanical mechanism in this paper, which we hope the reviewer would understand. We have just mention this future direction in the “limitations of the present study” (ll. 381 - 394).

Page 03, lines 24-26: Please, cite a bibliographic reference.
We modified the part and cite a reference (ll. 26 - 30).

Page 04, lines 49-51: It is not well defined that this adaptation may be due only to specific training of running. I suggest exploring this premise, based on physiomechanical studies. In addition, cite some references.
The word “adaptation” was vague and confusing. To make the logic straightforward, we have deleted this part.

Page 04, lines 53-54: This was not introduced. What do studies show about it? Specifically, between stride length and frequency?
We modified the prior sentence and changed “these individuals” to “these subjects” so as to make the meaning clearer. (ll. 50-51)

Page 04, lines 54-61: This is a methodological aspect (it should not be here).

The corresponding sentence was moved to the Method section.

Page 04, line 64: What is the concept of efficiency adopted here? Metabolic or Mechanical?
Thank you for the question. In this sentence, "efficiently" includes not only metabolic efficiency but also mechanical efficiency, because saving energy in any possible way would be especially important for distance runners. We have modified the part so it now reads as: “Among the four groups, the distance runners would be expected to run as efficiently (either mechanically or metabolically) as possible. As noted above, in the lower velocity ranges, altering stride length is a more energy saving strategy for changing velocity than is altering cadence” (ll. 51 - 54).

Page 04, lines 61-65: Based on which studies do the authors believe this?
From the aspect presented in the studies by Yanai and Hay (12), we hypothesized that distance runners should develop a more efficient way of running than the other groups.

Page 04, lines 66-71: This is a methodological aspect (it should not be here).
The corresponding sentence was moved to the methodology section and revised.

Methods: Some methodological questions are presented as a requirement for scientific acceptance.

Page 05, line 75: We know that there are physiomechanical differences between men and women runners. For example, the running economy. Was there any concern with sample homogenization in order to allow comparison between groups?
We appreciate the important comment. It might be necessary to consider gender differences in areas such as running economy. However, we were unable to recruit female sprinters or long-distance runners in our subject recruitment environment. Gender differences will be studied in the future, but since there seems no clear difference in the characteristics of the V-C-S relationship, we combined the data of male and female subjects together. (Please see supplemental data in Figures S3, and S4. Subjects with (F) are female).

Page 05, line 77: The subjects were classified into 3 groups according to sports experience. However, it is known that physiomechanical changes in running occur for physiological reasons, too. The physical conditioning was homogenized between the groups? Also, what are the specific parameters (in particular background)?
We appreciate the pointing out important issues. Different types of sports would lead to different physical changes, such as height. The main focus of the present study is “running-specific training to run faster”, which is the specific background considered. Therefore, in this study, subjects having experiences in other sports than sprinting and distance running were grouped together as “active athletes”.

Page 05, line 80: All
We revised it.

Page 05, line 82-84: Here I have some considerations to make. Some studies have demonstrated the influence of the sports motor gesture on the running technique. For example, studies with Triathlon. In addition, the way of carrying body mass can result in different physiomechanical behaviors of running (see allometric studies - Brisswalter, Kruel, etc ...). Why judo, volleyball, basketball, rowing, etc... All of these have different motor characteristics!
We appreciate the important suggestion. We are also interested in differences in the V-C-S relationship depending on the demands of various kind of sports. As noted above, our main focus of this study was the effect of “running-specific training for running faster”. As the first step, we have grouped together all active athletes of different sports other than sprinting and distance running. Interestingly, individual differences in the V-C-S relationship in active athletes were not particularly different from those of other groups (for example, Figs. 3 and 4). This suggests a stronger influence of the running-specific training relative to other factors. We and others may need to examine the detailed characteristics of each particular sport in future studies.

Page 06, line 95: Table 1: I believe that for the sampling characterization the maximal, aerobic, anaerobic capacities, and the leg length, should be informed. For age, I suggest adopt values without decimal places (mean and SD). Also specify the training volume.
We appreciate the important suggestion. Unfortunately, we have not measured such parameters as the reviewer listed. This is. of course, a limitation of this study. Yes, we are very interested in doing an analysis of the effects of these parameters in future studies. We emphasized this limitation in the Discussion (ll. 381 - 394).

Page 06, line 102: Why 30m?

We set this distance to get reliable average values over a particular interval. Of course the larger the number of steps used for the calculation, the more reliable it is. On the other hand, the subjects had to run many times, so we also had to take fatigue into account. After doing several preliminary experiments, we decided to employ 20m - 30m.

Page 06, lines 102-103: Why 20m for the subjects of the sedentary group?
We set 20 m for the sedentary group in consideration of their physical strength and lack of stamina. Please also see the note above.

Page 06, line 109: Why 60 Hz? Normally, frequencies from 120 Hz are adopted, mainly in the case of sprinter runners...

Thanks for your comment. Unfortunately, only video cameras with 60Hz were available for this study. Certainly, a high-speed camera could have provided more information. However, even with 60 Hz, for our particular analysis no important information would have been lost because the objects of our measurement were not exceptionally fast.

Page 06, lines 110-112: Was any subjective adaptation of effort made prior to the performance?
Since the subjects ran at various running speeds many times, we did not set up any special adaptation trials. Subjects began the experiment just after warming up.

Page 08, line 157: Figure 1: This is a result aspect (it should not be here). Also, there is no need for this information graphically.

We appreciate the suggestion. In this study, it is important to help readers understand the regression analysis and the concept of the inflection point correctly. Therefore, we did not omit Figure 1 and rather moved it to the Result section.

Page 09, line 167: Figure?

It is "Figure S4". We revised the sentence.

Page 09, lines 165-173: This paragraph is not necessary.
We think it is necessary to explain which subjects were excluded and why. We simplified it, so it now reads: “Subjects with inflection points, thus obtained, that differed largely from the measured points, were excluded from the analysis (#18, #19, and #20, as seen in Figure S4)” (ll. 148 – 149).

Page 09, lines 176-178: Was a sample calculation performed? And a homogeneity test?
We performed Levene’s Test of the SPSS.

Page 09, lines 178-180: Why haven't parametric transformation tests been adopted? Non-parametric analyzes adopt median values, which can contribute to the existence of statistical errors.
As a statistical method, it is common to perform non-parametric analysis on variables with a non-normal distribution. Therefore, we adopted the non-parametric analysis.

Page 10, line 189: Please, adopt p in italics.

We made the revision; p is now in italics.

Results: There is a lot of information in each figure. Furthermore, the figures are not self-explanatory. Finally, the figures are not of good quality. I suggest that the authors adopt tables of results or detailing (in paragraphs) the main results of each set of results.
We apologize for the bad quality of the figures. We hope they are now much better. We have revised the explanation of each figure to make then all as clear as possible. We added Table 2 to provide numerical data concerning the inflection point.

Page 10, lines 193-197: The Table 1 has already been presented. This information is redundant.

We deleted the part.

Page 10, lines 198-201: The Figure 1 has already been presented. This information is redundant. The quality of the figure is not good.

We moved the Figure 1 from the Method section as suggested by another reviewer. Therefore, the explanation of Figure 1 was left here.

Page 10, line 202: Figure 2: I suggest adopting a data table or writing the results in a paragraph.
The old Figure 2 was redundant with Fig. 3 and a supplementary figure. Therefore, we deleted it.

Page 11, line 219: These figures refer to a lot of information. It is suggested to adopt a paragraph highlighting the most important information.

I deleted the paragraph because it overlapped with the explanation in the Method section.

Discussion: Very speculative.
We appreciated the comment. We have modified the whole Discussion, and hope that it is now less speculative and more based on clear logic.

Page 14, lines 292-294: Figure 2: Difficult to perceive this visually.
We removed the old Figure 2 to make the manuscript simpler and easier to read.

Page 14, lines 295-297: This changes in step length and cadence are justified why? A discussion is needed!
We apologize that the explanation was too simple. We have revised the part to now read: “In all four subject groups analyzed an abrupt change in the V-C-S relationship took place at the inflection point (Fig. 3, and Table 2); a given velocity change occurred mainly due to a change in step length and cadence below and above the inflection point, respectively. These characteristics were demonstrated in preceding studies conducted on sprinters and distance runners (7, 9), and are particularly prominent in sprinters.” (ll. 302 - 306). We hope the part is now easier to understand.

Page 15, lines 299-300: In this topic, there is no discussion of the results with the literature. There is a methodological justification! A physiomechanical methodological discussion of the results is not demonstrated.
We certainly agree with your opinion. This section included no special information, and was thus deleted.

Page 16, lines 332-333: The discussion is very descriptive and superficial. Physiomechanical aspects are not discussed, which could enrich the scientific contributions of the present study. An approach to neuromuscular and physiomechanical studies of running is suggested.
We appreciate the very important suggestion. As the reviewer pointed out, it would be worthwhile to explain the results of this study in terms of neuromuscular and physiomechanical point of view. However, we are afraid that such discussion would became too speculative, since we have not obtained any neuromuscular or physiomtchanical data in this study. Therefore, we only briefly mentioned them in the limitation section (ll. 381 - 394). They are certainly the topic of future studies.

Pages 16-17, lines 344-348: This discussion is very superficial.
We have added the explanation on what the basic V-C-S relationship is (ll. 302 - 309).

We are regretful that we can make only a superficial discussion, because, as noted above, we have no data available to support or turn down any neuronal or physiomechanical possibilities. However, we are confident about the quantitative analysis of the present study. We are sure that this study will provide a good foundation for future studies that analyze the neuronal/physiomechanical mechanisms that are involved.

Page 19, line 392: This topic is very speculative.
We have completely modified this part, trying to make it least speculative.

Page 21, line 453: These limitations make the study quite superficial. Studies with a greater control of intervening parameters are suggested.

We appreciate the suggestion. We are planning to do such studies.

Page 22, line 467: There is no objective conclusion regarding the influence of sports motor characteristics (independent variables) on the dependent variables investigated in the present study. Again, there is a description of results.

We appreciate the suggestion. We have totally modified the conclusion so that it now reads as: “In the present study we analyzed the V-C-S relationship of running with the segmented regression method and made a quantitative comparison of the “spatiotemporal running characteristics” in subjects with different histories of running-specific training. The common characteristic of the V-C-S relationship is, in the lower and higher velocity ranges, that velocity is mainly modulated by altering step length and cadence, respectively, This was observed not only in the sprinters and distance runners, as shown in previous studies, but in active (general sport) athletes and sedentary subjects as well. In spite of the wide range of sports, the running modalities of the subjects, and their maximum running velocity, the inflection point appeared at a similar cadence (3.0 ± 0.2 steps/s) and at similar a relative velocity (65-70%Vmax), across all groups. These results imply that the influence of running-specific training on the inflection point is minimal” (ll. 396 – 407) We hope that the revision will provide an objective conclusion.

Page 23, line 480: 11% references between 2021 and 2016; 59% between 2015 and 2000; 30% before 2015.

We have added some references.

- **Reviewer #3:**

This manuscript presents interesting and novel findings. There may be broader implications, that can be investigated in the future, i.e. WHY is the critical velocity so consistent? What is being optimized? The study was simple but well-executed.

We appreciate the positive evaluation of the paper. We have revised the manuscript according to your and other reviewers’ suggestions. We agree that the former version contained too many speculative descriptions and also that the structure was overly complicated. Therefore, we deleted Fig. 2, showing the V-C-S curve for all the subjects in one diagram, because the basic messages overlap with Fig. 3 and the supplementary figures. We added Table 2 to provide numerical data concerning the inflection point. Finally, we changed the order of Fig. 3 (new Fig. 3) and Fig. 4 (new Fig. 2). We hope that it has been satisfactorily revised relative to the old version, and the the logic has become clearer and more straightforward.

Major points:

1. Title: For such an interesting paper, the title is a bit boring, vague and not informative.
Suggestion: “No effect of running-specific training on the velocity at the step length-cadence inflection point”
Another reviewer also suggested changing the title. We modified it so that it now reads: “Spatiotemporal inflection points in human running: effects of training level and athletic modality”.

2. Line 108 Can the authors provide any evidence that the subjects ran the 20/30 m at a constant velocity and were not accelerating/decelerating?
We appreciate the question. It is actually difficult to provide this evidence. Therefore, we took the average running velocity, SL, and SF as representative values for each trial.

3. Line 106 “additional 10-30 meters” is too vague. Was it always 10, always 30, variable between subjects? Few if any sprinters reach maximum velocity in < 30m. this does not affect main conclusion but authors should note that max in this study is probably not maximum.

We appreciate the suggestion. We did not specify the distance for acceleration and did not measure the acceleration distance for each trial. We added this point in the method section, which now reads as: “An additional 10-30 meters was provided before and after the filming zone (of 30m or 20m) so that the subjects could accelerate and decelerate and thus maintain running velocity as constant as possible throughout the recording area. This acceleration distance differed in each trial and was selected by the subject.” (ll. 90 - 94) As you pointed out, the maximal running velocity thus measured may not be the true maximum.

Minor Points:
Throughout the entire manuscript try to use “faster” or “slower” when describing velocity, not “higher” and “lower”

Thanks for the suggestion. We revised all of them accordingly.

Perhaps PLOS One does not require it but I would be interested to know the contributions from each of the EIGHT authors. I am also curious as to why Sony is interested in this topic!
We had been working with Sony on a different project. We tried to develop an apparatus that gave amateur joggers special rhythms so that they could modify their running pattern (especially cadence). However, we failed! As we now know retrospectively, cadence cannot be controlled in the lower velocity range at which joggers run. Actually, it is the starting point of this experiment to analyze basic V-C-S relationship across a wide range of subjects. The Sony co-authors in the study are scientists so that they are interested in the topic and joined in the experiments, especially in the data analysis.

Line 6 Group average maximal running velocities ranked from fastest to slowest were: sprinters, distance runners….

We corrected it.

Line 9 ‘as running velocity increased…”

We changed the sentence so that it now reads as: “In the velocity ranges below and above the inflection point (slower and faster velocity ranges), velocity was modulated primarily by altering step length and by altering cadence, respectively. This pattern was commonly observed not only in sprinters and distance runners, as has already been reported (Weyand et al., 2000; Nummela et al., 2007), but also in active athletes and sedentary individuals. This has been suggested as an energy-saving strategy (Yanai and Hay, 2004).” (ll. 12 - 17)

Line 10 slower and faster than the inflection point,

Please see the above.

Line 12 sentence beginning “Since” should be moved to the final sentence of Abstract. Also, add: “…is minimal, we surmise (or speculate or propose) the basic pattern is largely innate.”

We appreciate the suggestion. For the revised manuscript we tried not to use the word “innate”, since it is too speculative. Therefore, we modified the final sentence to now read as: “In spite of the wide range of athletic modalities of the subjects, as well as their maximum running velocities, the inflection point appeared at a similar cadence (3.0 ± 0.2 steps/s) and at a similar relative velocity (65-70%Vmax), across all groups. These results imply that the influence of running-specific training on the inflection point is minimal.” (ll. 19 - 23)

Line 16 …the inflection point was not different between groups

We changed the sentence accordingly.

Line 17 “frequency of running” here could be confused with cadence. I suggest: “Thus, the type, degree and frequency of running training had no effect.”
We changed the sentence accordingly.

Line 24 suggestion, replace much of lines 24-30 with: “As a matter of fact, velocity equals the product of cadence and step length. But the relative contribution of each component to changing velocity differs across the velocity range. At slower velocities, speed is modulated primarily by adjusting step length whereas at faster velocities, speed is modulated more by changes in cadence (6).”

Thanks for the good suggestion. We revised the text accordingly.

Line 34 “the relative contributions of cadence and step length optimize power production….”
We changed the sentence accordingly.

Line 41+ is a mega paragraph. To communicate more clearly, I urge the authors to cut this into several smaller paragraphs, each with a topic sentence. E.g. break at line 51 “Therefore, the purpose…

We appreciated the suggestion. Accordingly, we divided the paragraph mentioned into two paragraphs. Furthermore, the first paragraph was complicated and difficult to understand, because the effect of training and adaptation were mixed. We removed the explanation about adaptation. We hope that this part has become straightforward in logic. (ll. 41 - 57)

Lines 44-47 can you provide a reference to support this statement?
As noted above we have deleted this part.

Line 52 “investigate” rather than “reveal”
We revised it.

Line 56-61 This belongs in the Methods section
We moved this part to the Methods section and it now reads as: “the purpose of the present study was to investigate how a change in running velocity altered the spatiotemporal adjustment between step length and cadence in subjects with different histories of engagement in running training; that is, (1) sprinters, (2) distance runners, (3) active athletes who had received no running-specific training, and (4) sedentary, untrained subjects.” (ll. 45 - 49)

Line 66 …established the critical velocity…” not “point”

We deleted sentence.

Line 80 … in many of the sports (comma) all subjects…
We corrected the sentence.

Line 83 capitalize American
We corrected it.

Line 102 (only 20m for the sedentary…
We corrected it.

Line 103 on which
We corrected it.

Line 109 specify “video sampling frequency”
It was 60 Hz, and we specified it.

Line 119 …each trial ranged from 30 seconds to 5 minutes
We modified the sentence so it now reads as: “The interval between each trial ranged from 30 seconds to 5 minutes, depending on the speed of the previous trial.” (ll. 104 - 105)

Line 124+ again, this is a mega paragraph. You should break it into several smaller paragraphs
We appreciate the suggestion. We divided the paragraph mentioned into two paragraphs at line 125

Line 129 end point (not goal point)
We corrected it.

Line 130 cut (steps)
We corrected it.

Line 131 cut (sec)
We corrected the sentence so it now reads as: “Mean cadence (steps/sec) was calculated by dividing the number of steps with the time taken to run over distance used by the steps.”. (ll. 114 - 116)

Line 133 end not “goal”
We corrected it.

Line 134 also expressed as the dimensionless ratio (depicted = a picture)
We changed 'depicted as' to 'expressed as', since it fits the usage better.

Line 138 analyses (plural)
We corrected it.

Line 167-168 I don’t understand. How can their data be inordinately low compared to their own data???

We have changed the sentence so it now reads as: “Subjects with inflection points, thus obtained, that differed largely from the measured points, were excluded from the analysis (#18, #19, and #20, as seen in Figure S4).”

Line 287 be concise: The present study investigated the relative… Or even better: We investigated the relative…
We corrected the sentence accordingly.

Lines 312-316 this is repeating what you already said earlier.

We deleted this sentence.

Lines 412=414 is too speculative. At least add “It may be” or ‘it might be possible that”

We appreciate the suggestion. We modified the sentence so it now reads as: “A higher velocity cannot be accomplished only with power, especially at the highest levels. It might be possible that sprinters need to maintain both power and economy to the upper limit, and inevitably and unintentionally develop mechanically efficient movements.” (ll. 350- 353)

Line 418 I do not understand this claim.

The sentence was too simple. We explained it more in detail, so that it now reads as: “Thus, distance runners might not necessarily have pursued only efficient movement (mechanical efficiency) to the limit, because much of their training time would be better spent for improving cardio-respiratory and metabolic functions. However, if this were the case, it could be beneficial to increase training on obtaining movements more like those of the sprinters in order to improve their overall performance.”

Lines 421-438 I urge the authors to cut this rambling speculation.

We have deleted the part. Instead we added a discussion concerning the step length in the range below the inflection point in lines 313 - 315.
