# Supplementary material for: Spatiotemporal inflection points in human running: Effects of training level and athletic modality
Source: PLoS One. 2021 Oct 18;16(10):e0258709. doi: 10.1371/journal.pone.0258709 (PMC8523042; doi:10.1371/journal.pone.0258709)
Supplement: Attachment — Submitted filename: Response to Reviewers_PONE-D-21-04779_Goto.docx [file pone.0258709.s006.docx]

Reviewer #1: Line 95-99: Subjects ran 30 times at speeds between 10-100%, so they ran about three times for each intensity (around that)!! That was my question in the previous comment. The suggestion only: I find it interesting to add that they ran more than once at roughly the same intensity – about 3x (even if it wasn't exactly the same as it was subjective. It is no a problem).

We appreciate the suggestion. We added a sentence as “However, this method did produce the necessary array of running speeds, and the subjects might run more than once at an intensity.” (ll. 101-102)

Line 321: Please, to review this phrase: “Scatter plots of all subjects of all the groups showed only a weak correlation between the Vmax and the velocity at the inflection point (Fig. 4D).” In fact, the weak correlation was the velocity at the inflection point normalized with Vmax, since absolute velocity had strong correlation (Fig 4A)

We appreciated the comment. We corrected it as suggested (ll. 320-322).

Line 32: space (“maximum .”)

We corrected it as “maximum (8).” (l. 31)

Line 138: space (“Deming regression .)

We corrected it as “Deming regression (23, 24).” (l. 139)

Reviewer #2: The changes made have substantially improved the manuscript. The authors agreed that the previous version contained too many speculative descriptions and also that the structure was too complicated. So, I recommend the publication of this manuscript in PLOS ONE.

Thank you for your comment and the recommendation.

The purpose of the present study was to investigate how a change in running velocity changes the spatiotemporal adjustment between step length and cadence in subjects with different histories of engagement in running training. The main finding is that the inflection point appeared at a similar cadence and at similar a relative velocity, across all groups. These results imply that the influence of running-specific training on the inflection point is minimal.

We appreciated the comment.

The authors agreed that the previous version contained too many speculative descriptions and also that the structure was too complicated. The Fig. 2 (which showed the V-C-S curve of all subjects in a diagram) was excluded as the basic messages overlapped with Fig. 3 and the supplementary figures. Was added Table 2 to provide numerical data on the inflection point. Finally, the order of Fig. 3 (new Fig. 3) and Fig. 4 (new Fig. 2) has been changed. The changes mentioned, accompanied by the changes in the text, made the study acceptable for publication.

We appreciated the comment.

Abstract: The authors have modified the abstract so as to be minimally speculative. It is suggested that references are not cited (page 2, lines 15 and 17).

I deleted references.

Introduction: This topic has been substantially improved and the authors also clarified some issues. In fact, authors have not done any physiological or biomechanical measurement in this study, just focusing on velocity, SF, and SL. The introduction presents relationships between justification, problem and scientific objective.

Methods: The changes made, resulting from some suggestions from the reviewers, improved the understanding of the methodological aspects adopted. The main focus of the present study was the “running-specific training to run faster", which is the specific background considered.

Results: The results were properly adjusted in the text. Authors have revised the explanation of each figure to make then all as clear as possible. Was added Table 2 to provide numerical data concerning the inflection point.

Discussion: The modifications were pertinent. Now, I am confident about the quantitative analysis of the present study. I am, too, sure that this study will provide a good foundation for future studies that analyze the neuronal/physiomechanical mechanisms that are involved.

Limitations of the present study: Very important!

Conclusions: Pertinent!

References: Authors have added some references.

We appreciated all the comment above.

Reviewer #3: The manuscript is much improved.

My only major suggestion is to delete Lines 350-379. I find this all too speculative. The study itself is solid and obviously the investigators are very excited to explore the topic further. But, in my opinion, the excessive speculation without data detracts rather than positively adds to the manuscript.

Thank you for the important suggestion. As suggested we deleted most of the part, and left just “A higher velocity cannot be accomplished only with power, especially at the highest levels. Sprinters would develop both power and economy to the upper limit, and inevitably and unintentionally develop mechanically efficient movements.” (ll. 349-352), because without this the paragraph becomes left unfinished.

Minor

Line 11 consider “critical velocity” rather than critical point

We corrected it as suggested (l. 11).

Line 14 This pattern was commonly observed in all four groups. not only in sprinters and distance runners, as has already been reported (Weyand et al., 2000; Nummela et al., 2007), but also in active athletes and sedentary individuals.

Thank you for your suggestion. We revised the part as suggested it (ll. 14-16).

Line 16 This pattern may reflect an energy saving strategy (Yanni & Hay, 2004).

We revised the sentence as suggested (l. 16).

Line 19 … wide variety of athletic experience of the subjects…

We revised the part as suggested (l.19).

Line 47…. In running training. Namely, we studied: 1. sprinters, 2. distance runners…

We revised the part as suggested (ll. 46-48).

Line 53 slower velocity ranges

We corrected it (l. 52).

Line 54. Therefore, we hypothesized: 1. the running step length/cadence patterns of individuals would be influenced by their running training experience and overall physical activity levels and 2. Distance runners would…

We revised the part as suggested (ll. 53-57).

Line 63 They were assigned into one of four groups depending on their current/pervious running training.

We corrected the part as suggested (ll. 62-63).

Line 67 Although running is involved in many sports, all subjects…

We corrected the part as suggested (l. 67).

Line 72 without a history

We corrected it as suggested (l. 72).

Line 86 all-weather

We corrected it as suggested (l. 86).

Line 100 match the exact percentage

We corrected it as suggested (ll. 100-101).

Line 102 When running at the minimum velocity, subjects followed…

We corrected it as suggested (ll. 102-103).

Line 103 slowly

We corrected it (l. 103).

Line 104 interval between trials ranged

We corrected it (l. 105).

Line 113 The instant at which…

We corrected it (l. 114).

Line 114 identified from the position

We corrected it (l. 115).

Line 115 number of steps by the time taken to cover that distance. The number of steps…

We corrected it (l. 116-117).

Line 118 between the instant of first foot

We corrected it (l. 119).

Line 123 30 trials by each subject

We corrected it (l. 124).

Line 124 was designated as their maximal running velocity. (cut: of the subject)

We corrected it (ll. 124-125).

Line 125 start new paragraph here

We corrected it (l. 126).

Line 162 Next, post-hoc pairwise…

We corrected it (l. 163).

Line 189 …as the intersection point

We corrected it (l. 190).

Line 192 significant differences between the groups in terms of maximum running velocity

We corrected it (ll. 193-194).

Line 193 .. revealed that the maximal velocity of the sprinters was faster compared to all the other subject groups…

We corrected it (ll. 194 - 195).

Line 196 The distance runner group exhibited significantly faster…

We corrected it (ll. 196 - 197).

Line 198 …absolute step length and step length normalized to height.

We corrected it (l. 199).

Line 207 open circles indicate each individual subject

We corrected it (l. 208).

Line 212 both absolute velocity and velocity normalized to maximal running velocity

We corrected it (ll. 212-213).

Line 255 Table 2 Kinematic variables at the inflection point

We corrected it (l. 256).

Line 266 approximately constant

We corrected it (ll. 267-268).

Line 274 … (Vmax) and: running velocity (A), cadence (B)…

We corrected it (ll. 274-276).

Line 288 As expected, compared to the sprinters, maximal running velocities were progressively slower in the distance runner, active athlete and sedentary groups.

We corrected it (ll. 289-290).

Line 293 Among the subject groups, the sprinters were the tallest and the sedentary group was the shortest.

We corrected it (ll. 294-295).

Line 294 … with Vmax was well-preserved …

We corrected it (l. 295).

Line 297 and longer steps

We corrected it (l. 298).

Line 299 It appears that a slower cadence would have required …

We corrected it (l. 300)

Line 302 In all four subject groups, an abrupt… Table 2). Velocity changes below the inflection point occurred mainly by modulating step length and velocity changes above the inflection point occurred mainly via cadence modulation.

We adopted it (ll. 302-305).

Line 307-309 cut this if you adopt my suggestions for line 302+

We deleted the part.

Line 310 …inflection point has a significant

We corrected it (l. 308).

Line 314 start a new paragraph here: Overall, regardless of training history…

We corrected it (l. 313).

Line 316 inflection not inflexion. Yes, they sound the same, but English is a weird language.

We corrected it (l. 316).

Line 316 remained

We corrected it (l. 317).

Line 330 …relationship are common across different subject groups

We corrected it (l. 329).

Line 331 …could be related to quality/quantity…

We corrected it (l. 330).

Line 335 …be expected for the…

We corrected it (l. 334).

Line 338 cut: “and performing”

We deleted it.

Line 339 It seems reasonable that some portion…

We corrected it (ll. 337-338).

Line 340 start new paragraph

We corrected it (l. 339).

Line 340 point also follows

We corrected it (l. 339).

Line 343 I don’t think that study actually MEASURED energy, they just postulate it

We revised the part as “In the velocity range below the inflection point, velocity change is mainly done with a change in step length; for energy-saving this is a more efficient strategy than is changing the cadence” (ll. 340-342).

Line 381 Sub-heading could be “Future Studies”

I changed the Sub-heading to “Future Studies” (l. 354).

Line 382-386 delete

We deleted the sentences except the first part “Why and by what means are there differences in the various parameters of the V-C-S relationship? In particular, the neural as well as physiomechanical mechanisms of differences in the V-C-S relationship should prove very interesting.” to be logically connected to the following sentence (ll. 355-357).

Line 388 I don’t understand this part about “modeling”

We revised the part as “Although numerical simulation of running and walking has many limitations (11, 12, 25), the differences in the V-C-S relationship could be analyzed with numerical models in terms of various energy costs.” (ll. 359-361). We hope now it is understandable.
