# Supplementary material for: Spatiotemporal inflection points in human running: Effects of training level and athletic modality
Source: PLoS One. 2021 Oct 18;16(10):e0258709. doi: 10.1371/journal.pone.0258709 (PMC8523042; doi:10.1371/journal.pone.0258709)
Supplement: Attachment — Submitted filename: Response to Reviewers_R3_PONE-D-21-04779.docx [file pone.0258709.s007.docx]

Academic Editor

Congrats, you have done a good work until now. As you can see below, just one reviewer has some minor points. However, I have some further suggestions to improve the clarity of text and figures. Also, I feel that your discussion is somewhat empty of rationale and references. Please, consider following carefully my recommendations as follows.

Please, pay special attention to your two abstracts (in the system and into the manuscript), the version in the system is not updated.

We corrected the abstract through the system of editorial manager.

In the first paragraph of introduction, consider including this work:

https://pubmed.ncbi.nlm.nih.gov/34197674/

We appreciate your introducing the very important reference. We added a sentence as “Mechanical approaches, such as Fenn's approach, have been used as useful tools to elucidate these energy cost determinants with many practical applications (14).” (ll. 33-35)

line 33 - consider including this reference in the list (5,11,12): https://doi.org/10.3389/fphys.2018.01789

We appreciate your suggestion. We added the recommended reference. (line 33)

line 39 - here, you uses references that didn't investigate that (specially 16). Consider including this classical work about this topic: https://pubmed.ncbi.nlm.nih.gov/9305998/ . Even, if you look carefully the figures of Cavagna et al., 1997, you'll see that 3Hz or very close (in slower speeds) at self-selected cadences.

We appreciate your suggestion. We deleted the reference (Moore, 2016). We added a sentence as “In addition, in the slower velocity range, Cavagna et al. (19) reported that preferred cadences take place in the proximity of 3 Hz.” (ll. 41-42)

line 342 - Consider including this reference showing that the stride length was associated with better running economy in distance runners　(<https://pubmed.ncbi.nlm.nih.gov/22978185/>)

We appreciate your suggestion. We added a sentence as “Indeed, it was shown that at 4.4 m/s velocity, in the range below the inflection point, the stride length was associated with better running economy in distance runners (27).” (ll. 347-349)

line 363 - consider including this review where the concept of efficiency is broadly explained (https://doi.org/10.3389/fphys.2018.01789).

We appreciate your suggestion. We added the recommended reference. (line 368)

line 364 - I suggest this writing:

This and also fatigue (https://pubmed.ncbi.nlm.nih.gov/26214838/), aging (https://pubmed.ncbi.nlm.nih.gov/18077249/ and https://pubmed.ncbi.nlm.nih.gov/27116643/) and gender (https://pubmed.ncbi.nlm.nih.gov/33064810/) differences, if any, are topics that merit future analysis.

We appreciate your suggestion. We revised the sentence as suggested. (ll. 369-370)

Figure 1 - consider removing the decimal scale (for example 4 instead 4.0) in the horizontal and iso-speed scales.

We corrected it as suggested.

Figure 2 - consider removing the decimal scale (for example 4 instead 4.0) in the vertical (A) and hor (B,C,D) scales.

We corrected it as suggested.

Figure 3 - consider removing the decimal scale (for example 4 instead 4.0) in the horizontal scale.

We corrected it as suggested.

Figure 4 - consider removing the decimal scale (for example 4 instead 4.0) in the vertical and hor scales.

We corrected it as suggested.

In addition, we corrected S1-S4 Figures (Supporting Information) in the same way as suggested above.

Reviewer #1: (No Response)

Reviewer #2: The changes made have substantially improved the manuscript.

I recommend the publication of this manuscript in PLOS ONE.

We appreciated the positive evaluation of our manuscript.

Reviewer #3: I have mostly minor comments, no need for me to review again (3rd time!?).

Thank you for checking the manuscript in detail with English correction.

Major

Lines 208 and 209 I think you mean Figures 2, not 4!

We revised it (ll. 211-212).

Liner 218 I think you mean Figure 3A not 2A

We revised it (line 221).

Minor

Line 31 velocity approaches the maximum

We corrected it (line 31).

Line 56 in the slower

We corrected it (line 59).

Line 61 add two commas: backgrounds, in terms of their running experience, participated

We corrected it (ll. 64-65).

Line 69 add colon. were: soccer

We corrected it (line 72).

Line 71 in their sport

We corrected it (line 74).

Line 93 differed between trials

We corrected it (line 96).

Line 114 The instants….. end points were

We corrected it (line 118).

Line 157 SPSS Statistics

We corrected it (line 160).

Line 159 test. Maximal running…

We corrected it (line 162).

Line 160 Plural. …to have non-normal distributions.

We corrected it (line 164).

Line 161 data for maximal

We corrected it (line 164).

Line 165 investigate the possible mechanisms responsible for the inflection point

We corrected it (ll. 168-169).

Line 175 was set at p < 0.05

We corrected it (line178).

Line 221 the velocities between

We corrected it (line 224).

Line 301 step lengths

We corrected it (line 304).

Line 319 inflection not “inflexion”

We corrected it (line 322).

Line 346 runners increased velocity by elongating both absolute step length (Figure 4C) and relative step length (Figure 4F) all the way to the upper running speed limit.

We corrected it (ll. 351-352).

Line 349 Obviously, maximal velocity…

We corrected it (line 354).

Line 350 A faster velocity

We corrected it (line 354).

Line 351 Sprinters need to develop

We corrected it (ll. 355-356).

Line 359 could help to answer our overall question.

We corrected it (ll. 363-364).

Line 363 Is the V-C-S pattern innate or does it develop…

We corrected it (ll. 368-369).

Line 364 also sex differences.

We corrected it (line 364).

Line 370 in the slower and faster

We corrected it (line 376).
